# Correction: The auditory representation of speech sounds in human motor cortex

**DOI:** 10.7554/eLife.17181

**Published:** 2016-04-27

**Authors:** Connie Cheung, Liberty S Hamilton, Keith Johnson, Edward F Chang

Cheung C, Hamilton LS, Johnson K, Chang EF. 2016. The auditory representation of speech sounds in human motor cortex. *eLife* **5**:e12577. doi: 10.7554/eLife.12577.

Published March 4, 2016

In the originally published article, author Liberty S Hamilton’s surname was incorrectly given as ‘Hamiton’. This has now been corrected.

The article has been updated accordingly.

